# Ethnoveterinary medicinal plants in Aseko District, Arsi Zone, southeastern Ethiopia

**DOI:** 10.1038/s41598-026-53207-x

**Published:** 2026-05-29

**Authors:** Jewar Safeno Jara, Melesse Maryo, Zerihun Girma

**Affiliations:** 1 Department of General Forestry, Mizan Tepi University, P.O. Box 260, Mizan Aman, Ethiopia; 2 Ethiopian Wild LIfe Conservation Authority, Home-Based at Ethiopian Civil Service University, Addis Ababa, Ethiopia; 3 Wondo Genet College of Forestry and Natural Resources, Hawassa University, Shashemene, Ethiopia

**Keywords:** Ethnoveterinary medicine, Medicinal plants, Indigenous knowledge, Livestock health, Aseko District, Ethiopia, Diseases, Ecology, Ecology, Health care, Plant sciences

## Abstract

Ethnoveterinary medicine remains a key component of livestock healthcare in rural Ethiopia, particularly in areas with limited access to modern veterinary services. This study documented ethnoveterinary medicinal plants, associated indigenous knowledge systems, and conservation challenges in Aseko District, Arsi Zone, southeastern Ethiopia, using a bilingual (English–Afaan Oromo) disease classification framework to improve diagnostic clarity and strengthen veterinary relevance. Data were collected from 182 informants across seven purposively selected kebeles representing highland, midland, and lowland agroecological zones. Semi-structured interviews, focus group discussions, guided field walks, preference ranking, direct matrix ranking, and local market surveys were employed following established ethnobotanical methods. Voucher specimens were collected, taxonomically authenticated, and deposited at the Ethiopian Biodiversity Institute (EBI) National Herbarium. A total of 66 ethnoveterinary medicinal plant species belonging to 49 families were documented. Herbs constituted the dominant growth form (42.4%), and most remedies were prepared from fresh plant materials harvested from wild habitats (66.7%). Leaves were the most frequently used plant part (46.5%), followed by roots and bark, while oral administration was the predominant route (67.5%). Gastrointestinal disorders, ecto-/endoparasitic infections, and dermatological diseases together accounted for 68.2% of total use reports. Informant Consensus Factor values ranged from 0.25 to 0.67, with the highest agreement observed for reproductive disorders, indicating relatively strong consensus among informants for specific ailment categories. Preference ranking identified *Hagenia abyssinica*, *Zingiber officinale*, and *Embelia schimperi* as the most preferred species for treating livestock diarrhoea. Despite high plant diversity, dependence on wild habitats combined with agricultural expansion and biomass extraction was identified as a major threat to long-term resource sustainability. The study demonstrates both the resilience and vulnerability of ethnoveterinary knowledge in a biodiverse but increasingly pressured landscape. Strengthening community-based conservation, systematic documentation of indigenous knowledge, and integration of validated ethnoveterinary practices into local livestock healthcare systems are strongly recommended.

**Supplementary Information:**

The online version contains supplementary material available at 10.1038/s41598-026-53207-x.

## Introduction

Ethiopia is widely recognized for its exceptional biological and cultural diversity, with rich flora and long-standing traditions of plant based healthcare^1,2^. Indigenous knowledge systems developed over generations have enabled rural communities to manage both human and livestock health using locally available medicinal plants, particularly in areas where access to modern veterinary services remains limited^3^. Livestock production plays a central role in Ethiopia’s mixed farming and pastoral systems, contributing substantially to food security, household income, draught power, and sociocultural identity^4^.

Despite extensive ethnobotanical and ethnoveterinary documentation across Ethiopia^8–10^, important geographical gaps remain. Many studies emphasize species inventories without systematically evaluating cultural importance, informant consensus, or perceived efficacy^16,17^. At the same time, traditional medicinal plant resources and associated knowledge systems are increasingly under pressure from agricultural expansion, deforestation, infrastructure development, and population growth^11–13^. Sociocultural change, formal education, and declining reliance on traditional practices have weakened intergenerational transmission of ethnoveterinary knowledge, particularly among younger community members^14,15^.

Aseko District in Arsi Zone represents one such gap. The district spans three agroecological zones (highland, midland, lowland) and supports high biodiversity but faces above average ecological pressure from agricultural expansion, fuelwood extraction, and limited veterinary infrastructure. These local threats make Aseko a critical site for documenting ethnoveterinary practices and assessing their sustainability. Importantly, this study introduces a bilingual disease classification system (English/Afaan Oromoo) to reduce diagnostic ambiguity and strengthen alignment between indigenous knowledge and veterinary science—a methodological advance over previous Ethiopian studies that relied solely on vernacular or clinical terminology^9,13,17^.

Among the reported livestock ailments, diarrhoea was the most prevalent and frequently treated condition in the study area. By generating detailed and locally grounded evidence, this study aims to contribute baseline information for biodiversity conservation, sustainable use of medicinal plants, and future ethnoveterinary research in Ethiopia.

Accordingly, the study was designed to: (i) comprehensively document medicinal plant species used to treat livestock ailments in Aseko District; (ii) record associated knowledge on plant parts used, preparation methods, routes of administration, and dosage practices; (iii) quantitatively evaluate informant agreement and cultural importance using established ethnobotanical indices such as Informant Consensus Factor (ICF) and Fidelity Level (FL)^18–20^; (iv) assess local perceptions regarding ecological threats, and conservation of ethnoveterinary medicinal plants. *(v) document local market integration*, and conservation priorities for *ethnoveterinary* medicinal plants;*species and* (v) evaluate the *perceived* therapeutic efficacy of antidiarrheal species through preference ranking, given that gastrointestinal disorders represent the largest ailment category (28.5% of use reports) and diarrhea is a leading cause of morbidity and economic loss in Ethiopian smallholder systems. By generating detailed and locally grounded evidence, this study aims to contribute baseline information for biodiversity conservation, sustainable use of medicinal plants, and future ethnoveterinary research in Ethiopia.*threats to their sustainability.*

## Results

### Informant demographics

A total of 182 informants participated in the study, comprising 30 key informants (16.5%) and 152 household respondents (83.5%) selected from seven kebeles of Aseko District. Most participants were male (80.2%, *n* = 146), while females accounted for 19.8% (*n* = 36). Ages ranged from 28 to 96 years (mean ± SD: 56.4 ± 14.8 years), with the largest proportion of respondents in the 51–65 year age group (34.6%), followed by 36–50 years (29.7%), > 65 years (23.6%), and 20–35 years (12.1%).

Older respondents (51–65 years and > 65 years) reported a greater number of medicinal plant species than younger respondents (20–35 years), indicating that ethnoveterinary knowledge is concentrated among older community members and suggesting potential intergenerational erosion of traditional knowledge.

The majority of informants were unable to read and write (65.4%), while 20.9% had elementary education, 9.3% had secondary education, and 4.4% had college-level training. Most respondents were Muslim (72.0%), followed by Christians (28.0%). Oromo (85.7%) and Amhara (14.3%) were the only ethnic groups identified during the survey.

Ethnoveterinary knowledge was transmitted mainly through family lineage (61.0%), followed by traditional healers (18.1%), religious learning (11.0%), and friends or relatives (9.9%), highlighting the dominant role of oral intergenerational knowledge transfer. No formal statistical tests (e.g., ANOVA or regression analysis) were conducted to evaluate the significance of these differences; therefore, observed patterns are interpreted descriptively rather than inferentially.

### Diversity of ethnoveterinary medicinal plants

A total of 66 ethnoveterinary medicinal plant species belonging to 62 genera and 49 families were documented in the study area (Table 1). Euphorbiaceae and Solanaceae were the most represented families, each contributing five species. The remaining families were represented by three or fewer species, with 40 of the 49 families (81.6%) represented by only a single species. This reflects high taxonomic diversity and a broad range of evolutionary lineages rather than concentration within a few dominant families.

Voucher specimens of all recorded species were authenticated and deposited at the Ethiopian Biodiversity Institute (EBI) National Herbarium, Addis Ababa. Corresponding voucher accession numbers and collector identifiers are provided in Supplementary Table S1 to ensure taxonomic traceability and future scientific verification.

**Table 1:** Diversity of ethnoveterinary medicinal plants documented in Aseko District.

Scientific name	Family	Local name	GF	Hb	PU
*Achyranthes aspera* L.	Amaranthaceae	Darguu	H	W	L
*Aeonium leucoblepharum* Webb ex A. Rich.	Crassulaceae	Harmaa gurraatti	S	W	L
*Agave sisalana* Perrine	Agavaceae	Algee	H	C	R
*Aloe vera* L.	Asphodelaceae	Hargiisa	H	C	L
*Allium sativum* L.	Amaryllidaceae	Qullubii-Adii	H	C	Bu
*Anethum graveolens* L.	Apiaceae	Koomana	H	C	R
*Asparagus africanus* Lam.	Asparagaceae	Sariitii	C	W	L, R
*Brassica nigra* (L.) Koch	Brassicaceae	Siinaficaa	H	C	Se
*Brucea antidysenterica* J.F.Mill.	Simaroubaceae	Jiloolaafaa	T	W	R
*Calpurnia aurea* (Ait.) Benth.	Fabaceae	Cekaa	T	W	L, R, Ba
*Capsicum annuum* L.	Solanaceae	Barberee	H	C	Fu
*Carissa spinarum* L.	Apocynaceae	Hagamsa	S	W	R
*Capparis tomentosa* Lam.	Capparaceae	Harangamaa	S	W	R
*Catha edulis* (Vahl) Endl.	Celastraceae	Jimaa	T	C	L
*Cissampelos mucronata* A.Rich.	Menispermaceae	Bishaan qal’ee	C	W	L
*Clutia abyssinica* Jaub. & Spach	Euphorbiaceae	Ulee foonii	S	W	L, R
*Croton macrostachyus* Del.	Euphorbiaceae	Makanisaa	T	W	L, La
*Cucumis ficifolius* A.Rich.	Cucurbitaceae	Araddoo gurraatti	H	W	R, L
*Cucurbita pepo* L.	Cucurbitaceae	Dubbaa / Dhobba	H	C	R, L + Ba
*Cynodon dactylon* (L.) Pers.	Poaceae	Coqorsa	H	W	Wp
*Dodonaea angustifolia* L.f.	Sapindaceae	Iticha	S	W	L, Se
*Discopodium eremanthum* Chiov.	Solanaceae	Mararo	S	W	L
*Dovyalis abyssinica* A.Rich.	Salicaceae	Kooshimo	T	W	L
*Embelia schimperi* Vatke	Primulaceae	Gurraa / Qorqoro	C	W	R
*Erythrina brucei* Schweinf.	Fabaceae	Walleensu	T	W	L, Ba
*Eucalyptus globulus* Labill.	Myrtaceae	Bargamo adii	T	C	L
*Euphorbia abyssinica* J.F.Gmel.	Euphorbiaceae	Adaami	T	W	L, Ba
*Hagenia abyssinica* (Bruce) J.F.Gmel.	Rosaceae	Heexoo	T	W	L
*Heteromorpha arborescens* (Spreng.) Cham. & Schltdl.	Apiaceae	Aliyaanqaa	T	W	R
*Hordeum vulgare* L.	Poaceae	Garbuu	H	C	Se
*Hypericum revolutum* Vahl	Hypericaceae	Garambaa	S	W	L + R
*Juniperus procera* L.	Cupressaceae	Hindheessa	T	W	R
*Justicia schimperiana* (Hochst. ex Nees) T.Anders.	Acanthaceae	Dhumugaa / Sensel	S	W	L + R
*Lepidium sativum* L.	Brassicaceae	Fexo / Shuunfaa	H	C	Se
*Linum usitatissimum* L.	Linaceae	Talbaa	H	C	Se
*Maesa lanceolata* Forssk.	Primulaceae	Abbayyii	S	W	L, R, Fu
*Melia azedarach* L.	Meliaceae	Mim	T	C	Ba
*Musa × paradisiaca* L.	Musaceae	Muuzii	H	C	Sa
*Myrsine africana* L.	Primulaceae	Qacama	S	W	R
*Nigella sativa* L.	Ranunculaceae	Habsuda guracha	H	C	Se
*Nicotiana tabacum* L.	Solanaceae	Timbaho / Tamboo	H	C	L, R
*Ocimum lamiifolium* Hochst.	Lamiaceae	Damakasee	H	W	L
*Olinia rochetiana* A.Juss.	Oliniaceae	Gunaa	T	W	L
*Phoenix reclinata* Jacq.	Arecaceae	Meexxi	T	W	L + St
*Phytolacca dodecandra* L’Hér.	Phytolaccaceae	Handoodee	C	W	Ba, L + R
*Plantago lanceolata* L.	Plantaginaceae	—	H	W	R
*Podocarpus falcatus* (Thunb.) Mirb.	Podocarpaceae	Birbirsa	T	W	Ba
*Plumbago zeylanica* L.	Plumbaginaceae	Merxes	H	W	L, R
*Premna schimperi* Engl.	Lamiaceae	Urgessa	T	W	L, R
*Rhamnus prinoides* L’Hér.	Rhamnaceae	Geeshoo	S	W	L
*Ricinus communis* L.	Euphorbiaceae	Qobboo	S	W	R, L, Fu
*Rubus steudnerii* Schweinf.	Rosaceae	Dhukkoo	S	W	L
*Rumex nepalensis* Spreng.	Polygonaceae	Shaabbee	H	W	R
*Ruta chalepensis* L.	Rutaceae	Teenaadaama	S	C	L + Ba
*Solanum anguivi* L.	Solanaceae	Hidi-bude	S	W	Se
*Sorghum bicolor* (L.) Moench	Poaceae	Bisingaa caabbi	H	C	Se, R, L
*Syzygium guineense* var.	Myrtaceae	Baddessa	T	W	Ba
*Teclea nobilis* Del.	Rutaceae	Hadheesa	T	W	L, R
*Thymus schimperi* Ronniger	Lamiaceae	Xooshine	H	W	L
*Tragia cinerea* (Pax)	Euphorbiaceae	Laalessaa	H	W	L
*Trichocladus ellipticus* Eckl. & Zeyh.	Hamamelidaceae	Dhadhaa	T	W	L
*Urera hypselodendron* (A.Rich.) Wedd.	Urticaceae	Haliilaa	S	W	Wp
*Verbascum sinaiticum* Benth.	Scrophulariaceae	Gurra Harree	H	W	L
*Vernonia amygdalina* Del.	Asteraceae	Ebicha	S	W	L
*Withania somnifera* (L.) Dunal	Solanaceae	Gizaawa / Uunjoo	S	W	R
*Zingiber officinale* Roscoe	Zingiberaceae	Zinjibila	H	C	Rh

GF = growth form (H = herb, S = shrub, T = tree, C = climber); Hb = habitat (W = wild, C = cultivated); PU = plant part used (L = leaf, R = root, Ba = bark, Fu = fruit, Se = seed, Bu = bulb, Wp = whole plant, Sa = sap/latex, Rh = rhizome, St = stem; “+” indicates combined parts used in a single remedy).

### Growth forms and habitat sources

**Fig. 1:**
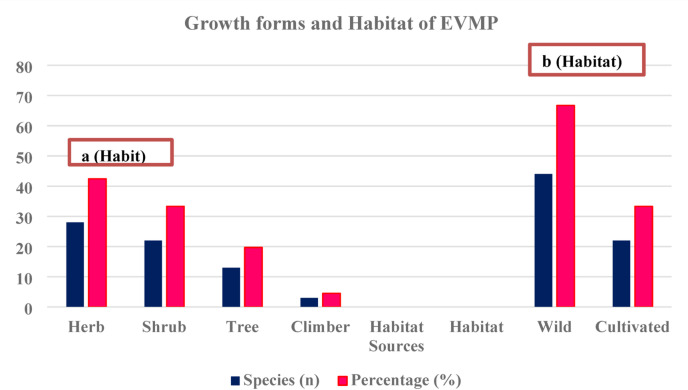
Growth forms of medicinal plants used in ethnoveterinary practices in Aseko District. Herbs constituted the dominant growth form (42.4%), followed by shrubs (33.3%), trees (19.7%), and climbers (4.5%).

Herbs were the dominant growth form (42.4%), followed by shrubs (33.3%), trees (19.7%), and climbers (4.5%) (Fig. 1). Most medicinal plants were harvested from wild habitats (66.7%), while the remaining 33.3% were obtained from home gardens, cultivated fields, and farm margins.

Informants consistently preferred fresh plant materials for remedy preparation. However, the strong dependence on wild habitats, combined with harvesting of roots (18.9%) and bark (10.7%), poses considerable conservation risks for slow-growing and habitat-restricted species.

### Livestock diseases and local terminology

A total of 33 livestock ailments were recorded using bilingual English and Afaan Oromoo disease classification (Supplementary Table S1). These included gastrointestinal disorders, parasitic infections, dermatological diseases, respiratory illnesses, reproductive complications, wounds and trauma, and ophthalmological/otic disorders.

The most frequently cited ailments were blackleg, diarrhea, bloat, and parasitic infestations. Blackleg (locally recognized and clinically corresponding to *Clostridium chauvoei* infection) was treated as a distinct major ailment category rather than being grouped under general gastrointestinal disorders, improving pathological clarity and strengthening disease classification accuracy.

The bilingual classification system improved interpretation of vernacular disease terms and reduced ambiguity in grouping ailments across informants.

### Plant parts used

**Fig. 2:**
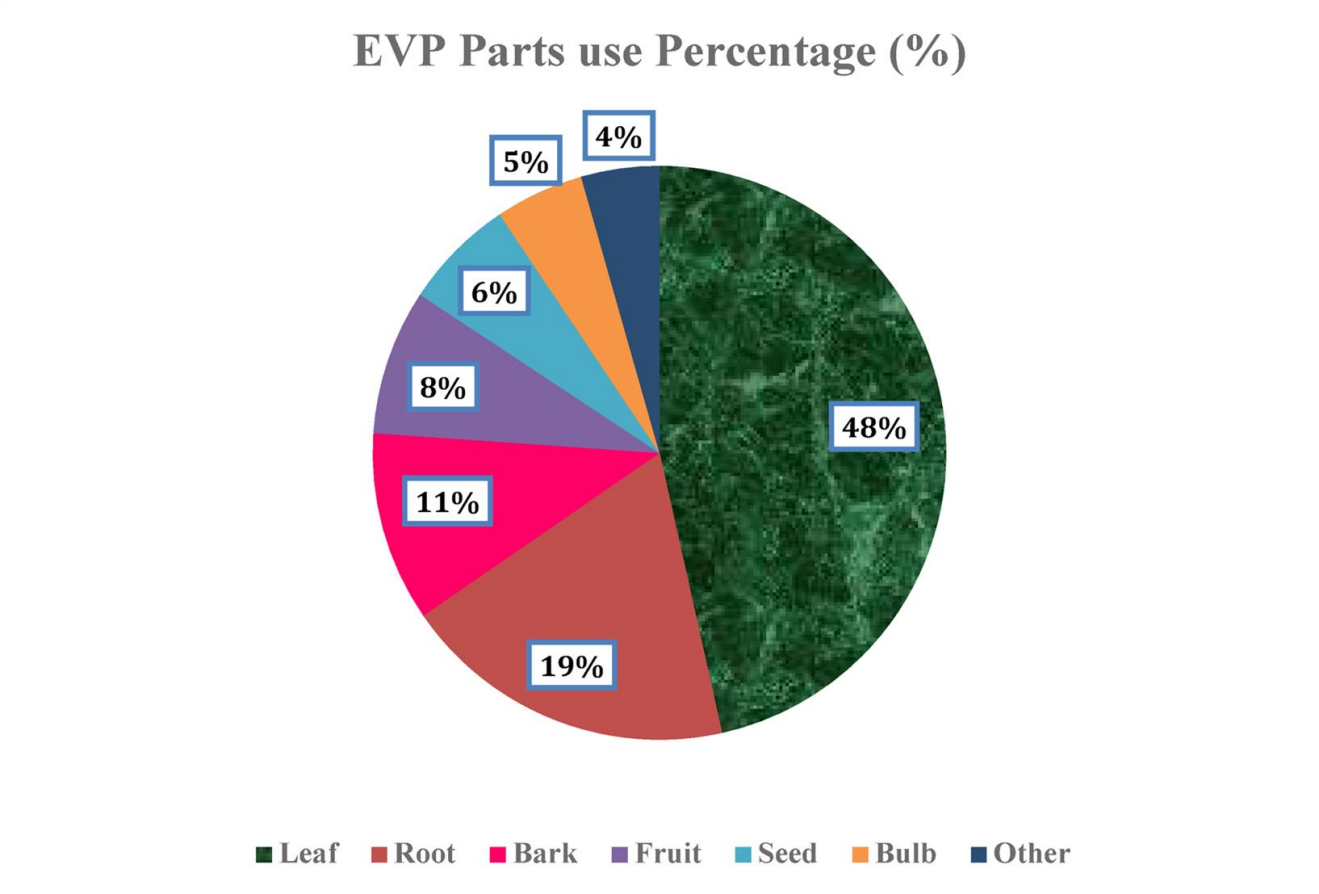
Percentage distribution of plant parts used for ethnoveterinary remedies in Aseko District. Leaves were the most frequently utilized plant part (46.5%), followed by roots (18.9%), bark (10.7%), fruits (8.2%), seeds (6.3%), bulbs (5.0%), and other parts including rhizomes, latex, and stems (4.4%).

Leaves were the most frequently used plant part (46.5%), followed by roots (18.9%), bark (10.7%), fruits (8.2%), seeds (6.3%), bulbs (5.0%), whole plants (1.9%), and other plant parts (2.5%) (Fig. 2).

Although leaf harvesting is generally considered less destructive, the relatively high use of roots and bark represents a major ecological concern because these practices are destructive and reduce plant regeneration capacity, particularly for wild populations.

### Modes of preparation

**Fig. 3:**
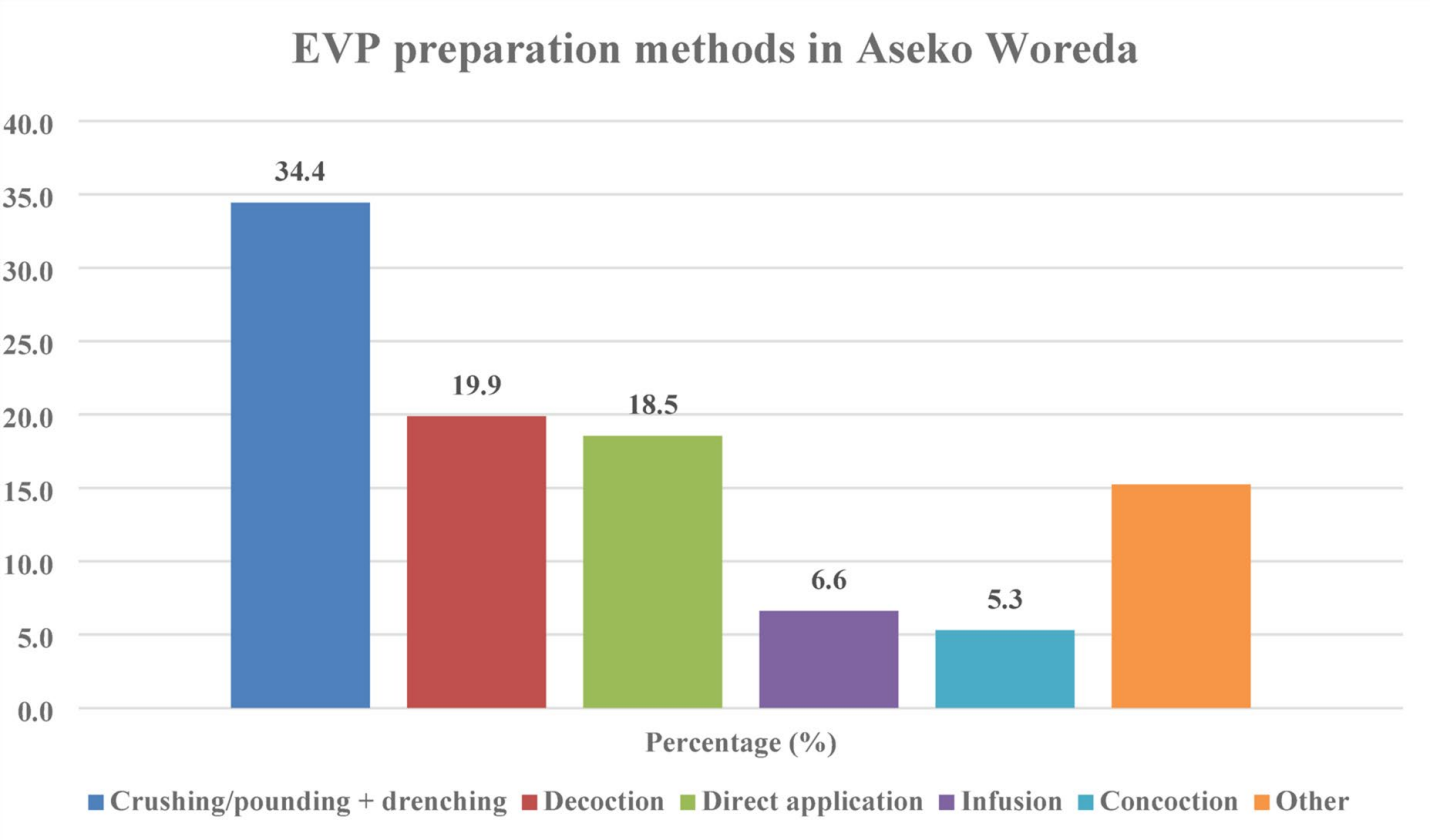
Common methods of remedy preparation used by traditional healers in Aseko District. Crushing or pounding followed by drenching was the most common method (34.4%), followed by decoction (19.9%) and direct topical application (18.5%).

Crushing or pounding followed by drenching was the most common preparation method (34.4%), followed by decoction (19.9%) and topical application (18.5%). Infusion (6.6%), concoction (5.3%), and crushing–pasting–tying (5.3%) were less frequently reported (Fig. 3).

In this study, “concoction” refers specifically to remedies prepared by combining multiple plant species, whereas “crushing” refers to the physical processing of a single plant species. These categories were therefore treated as mutually exclusive for analytical clarity.

### Routes of administration and dosage

**Fig. 4:**
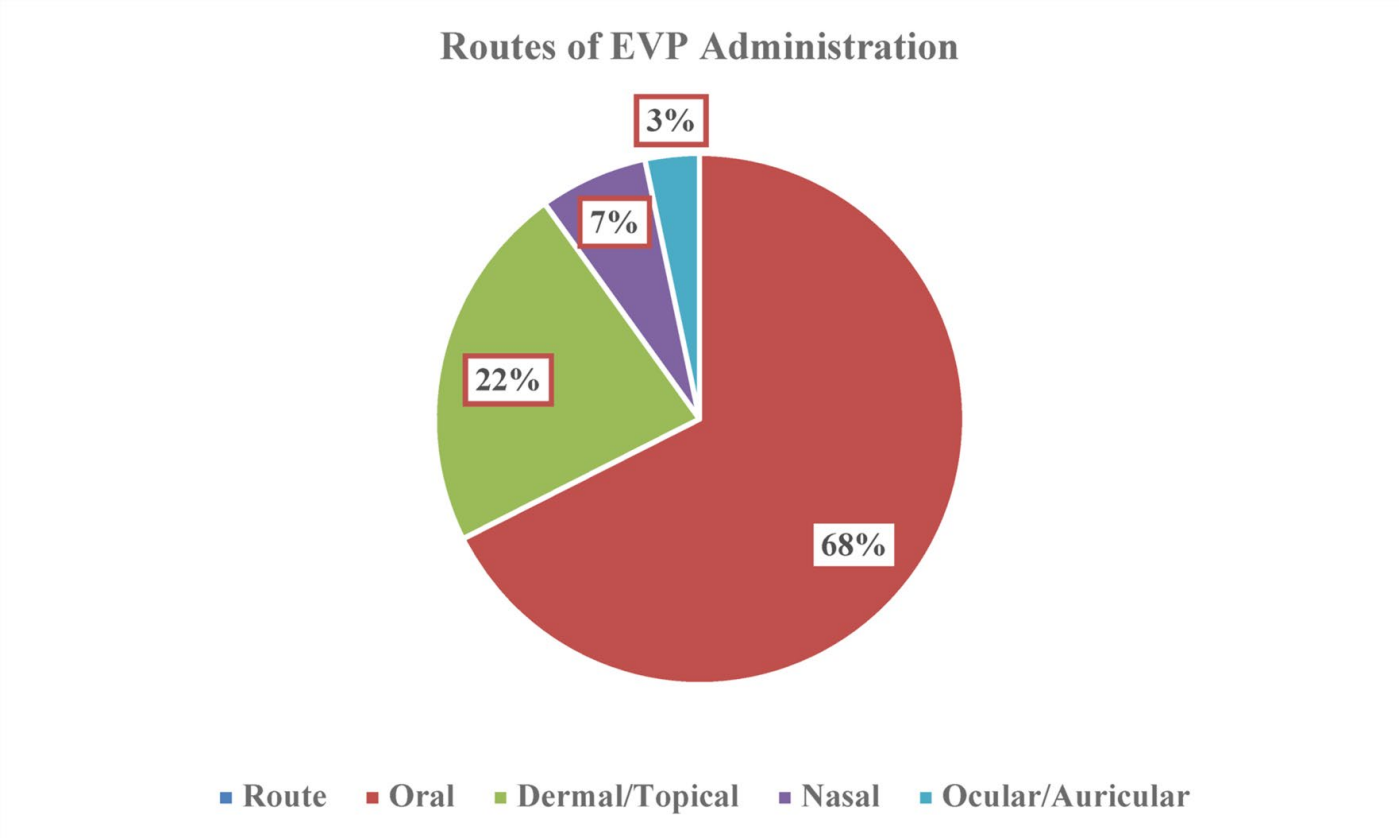
Major routes of administration of ethnoveterinary remedies in Aseko District. Oral administration was predominant (67.5%), followed by dermal/topical application (22.5%), nasal administration (6.6%), and ocular/auricular application (3.3%).

Oral administration was the dominant route (67.5%), followed by dermal application (22.5%), nasal administration (6.6%), and ocular/auricular application (3.3%) (Fig. 4).

Dosage was determined mainly by healer experience using local measurement units such as cups, bottles, spoons, handfuls, and finger lengths. Informants acknowledged variability in dosage and occasional treatment failure or side effects, reflecting the absence of standardized dosage systems and limited awareness of toxicity thresholds.

### Major categories of ailments

Gastrointestinal disorders accounted for the highest proportion of use reports (28.5%), followed by ecto-/endoparasitic infections (24.5%), dermatological diseases (15.2%), wounds and musculoskeletal trauma (13.2%), respiratory infections (7.3%), reproductive disorders (4.6%), ophthalmological/otic disorders (3.3%), and other conditions (3.3%) (Table 2).

The previously used term “sensorial ailments” was replaced with the standard veterinary category “ophthalmological/otic disorders” to improve scientific clarity and consistency.

**Table 2:** Ethnoveterinary use reports across livestock ailment categories. Summarizes the number of use reports, number of taxa, and Informant Consensus Factor (ICF) values for eight major veterinary ailment categories, with gastrointestinal disorders accounting for the highest proportion of use reports.

Ailment category	Use reports	Percentage (%)	Key species (Fidelity Level, %)
Gastrointestinal disorders	43	28.5	Embelia schimperi (92), Zingiber officinale (88)
Ecto-/endoparasitic infections	37	24.5	Phytolacca dodecandra (85), Calpurnia aurea (80)
Dermatological diseases	23	15.2	Croton macrostachyus (89), Allium sativum (85)
Wounds and musculoskeletal trauma	20	13.2	Asparagus africanus (78), Ricinus communis (75)
Respiratory infections	11	7.3	Ocimum lamiifolium (88), Cissampelos mucronata (73)
Reproductive disorders	7	4.6	Aeonium leucoblepharum (68)
Ophthalmological/Otic disorders	5	3.3	Trichocladus ellipticus (81)
Other conditions (sudden sickness, evil eye, urine retention, weakness)	5	3.3	Withania somnifera (71)
Total	151	100.0	

### Informant consensus factor (ICF)

ICF values ranged from 0.25 to 0.67 across ailment categories. The highest consensus was observed for reproductive disorders (ICF = 0.67), indicating moderate-to-high agreement among informants regarding the plants used for these conditions. Gastrointestinal disorders and wounds/trauma showed moderate consensus (0.55–0.58), while the “other conditions” category showed the lowest consensus (0.25).

These results suggest that strong shared ethnoveterinary knowledge exists only for specific ailment groups, whereas low ICF values may reflect variability in treatment practices or erosion of shared knowledge.

### Preference ranking

Preference ranking of medicinal plants used for treating livestock diarrhoea identified *Hagenia abyssinica* as the most preferred and perceived as the most effective species, followed by *Zingiber officinale* and *Embelia schimperi* (Table 3).

This ranking is based on scores assigned by key informants reflecting their experiential evaluation of therapeutic effectiveness.

These findings indicate that perceived therapeutic effectiveness does not always correspond directly with citation frequency, highlighting the importance of combining ranking methods with citation-based indices.

**Table 3:** Preference ranking of medicinal plants used to treat livestock diarrhea. Fourteen key informants assigned scores from 1 (least effective) to 7 (most effective) to seven antidiarrheal species; total scores determine rank order, with Hagenia abyssinica ranked first, followed by Zingiber officinale and Embelia schimperi.

Medicinal plant	Informant scores (A–*N*)	Total score	Rank
*Hagenia abyssinica* (Bruce) J.F.Gmel.	7, 6, 7, 5, 6, 7, 6, 7, 4, 6, 7, 7, 6, 5	86	1st
*Zingiber officinale* Roscoe	7, 4, 3, 2, 5, 6, 4, 2, 7, 3, 7, 5, 6, 7	68	2nd
*Embelia schimperi* Vatke	4, 5, 7, 5, 6, 3, 3, 6, 5, 4, 6, 3, 3, 5	65	3rd
*Clutia abyssinica* Jaub. & Spach	5, 2, 6, 6, 4, 3, 5, 6, 6, 5, 4, 2, 3, 4	61	4th
*Ocimum lamiifolium* Hochst.	4, 6, 4, 5, 2, 1, 4, 4, 5, 6, 5, 3, 2, 6	57	5th
*Rubus steudnerii* Schweinf.	3, 2, 1, 4, 5, 2, 1, 5, 4, 3, 5, 2, 3, 4	44	6th
*Dodonaea angustifolia* L.f.	1, 3, 2, 4, 3, 5, 1, 2, 4, 5, 4, 4, 3, 2	43	7th

Scores were assigned by 14 key informants using a scale of 1 (least effective) to 7 (most effective). Total scores represent cumulative perceived efficacy; higher values indicate stronger preference.

### Fidelity level (FL)

High Fidelity Level (FL) values were recorded for *Embelia schimperi* (92.3%), *Ocimum lamiifolium* (88.2%), and *Phytolacca dodecandra* (85.2%) Table 2), indicating strong ailment-specific use and cultural importance.

FL values were calculated using all 182 informants who reported medicinal plant use, while preference ranking involved only selected key informants. This distinction improves interpretive clarity and avoids ambiguity in denominator definition.

### Multipurpose use and conservation priority

Direct matrix ranking revealed that *Hagenia abyssinica*, *Croton macrostachyus*, *Podocarpus falcatus*, and *Juniperus procera* were under the highest multipurpose use pressure due to their simultaneous use for medicine, firewood, construction, fencing, tools, and shade (Table 4).

Such combined utilitarian and medicinal demand increases harvesting pressure and places these species at higher conservation priority.

**Table 4:** Direct matrix ranking of multipurpose ethnoveterinary medicinal plants in Aseko District. Twelve key informants scored eight widely used species across eight use categories (medicine, firewood, fodder, construction, food, fencing, tools, shade) on a 0–5 scale (0 = not used, 5 = highest utility); composite use values indicate species under greatest multipurpose harvest pressure.

Species	Medicine	Firewood	Fodder	Construction	Food	Fencing	Tools	Shade	Total use value
*Hagenia abyssinica*	4.8	3.2	2.1	4.5	1	3.7	2.9	4.3	26.5
*Ruta chalepensis*	4.5	2.8	1.9	4.2	0.8	3.5	2.7	4	24.4
*Croton macrostachyus*	4.3	3.9	2.6	3.8	1.2	3.4	2.5	3.1	24.8
*Juniperus procera*	4.1	4	1.5	4.7	0.5	2.8	3.6	2.9	24.1
*Podocarpus falcatus*	3.9	4.5	1.8	3.5	2	3	2.2	3.3	24.2
*Ocimum lamiifolium*	4.7	1.2	2.4	1	3.8	1.5	1	2.1	17.7
*Phytolacca dodecandra*	4.4	2	3.1	1.8	2.5	2.9	1.3	1.7	19.7
*Withania somnifera*	4.6	1.5	2.8	1.2	3	2	1.8	2	18.9

### Major threats to ethnoveterinary medicinal plants

Perceptions of local communities regarding threats to ethnoveterinary medicinal plants were assessed using a ranking exercise with seven key informants. The identified threats and their relative importance are presented in Table 5.

Agricultural expansion was identified as the most severe threat (22.0%), followed by fuelwood and charcoal production (19.8%). Additional pressures included overgrazing, uncontrolled harvesting for construction materials, and climate-related stresses such as drought and soil erosion.

These results indicate that medicinal plant resources in the study area are subject to multiple, interacting anthropogenic pressures linked to land-use change and livelihood dependency.

### Perceived threats to medicinal plant resources

Agricultural expansion was identified as the leading threat, followed by fuelwood extraction and climate-related stress.

**Table 5:** Major threats to medicinal plants in the study area. Threats were scored by seven senior traditional healers across six factors; percentages reflect aggregated scores, with agricultural expansion (22.0%) identified as the primary threat, followed by firewood and charcoal collection (19.8%).

Threat factor	Total score	% of total	Rank
Agricultural expansion	40	22.0	1st
Firewood & charcoal	36	19.8	2nd
Climate stress (drought/erosion)	34	18.7	3rd
Construction & timber	29	15.9	4th
Grazing pressure	27	14.8	5th
Pesticide use	22	12.1	6th

### Marketability

Fifteen species (22.7%) were found to be sold in local markets. Market surveys were conducted in Aseko, Chafe Donsa, and nearby weekly markets during regular market days, and prices were recorded using local sale units such as bundles, cups, bottles, and root portions.

Examples included *Withania somnifera* roots (approximately 30 Ethiopian Birr per bundle) and *Thymus schimperi* leaves (approximately 20 Ethiopian Birr per cup). The marketability of these species demonstrates their economic importance but may also contribute to overharvesting pressure.

### Knowledge transmission

Ethnoveterinary knowledge was transmitted almost entirely through oral communication, primarily within family lineages and often from elders to elder sons. Very limited written documentation existed at the household level, increasing vulnerability to knowledge loss as younger generations show declining participation.

## Discussion

This study documents a highly diverse ethnoveterinary knowledge system in Aseko District, encompassing 66 medicinal plant species distributed across 62 genera and 49 families. This substantial taxonomic breadth reflects strong reliance on plant-based livestock healthcare and indicates that local healers utilize a wide range of evolutionary plant lineages rather than a few culturally dominant taxa. The prominence of families such as Euphorbiaceae and Solanaceae, together with the high proportion of single-species families (81.6%), aligns with patterns reported from other parts of Ethiopia and East Africa^22,24,31^. Similar taxonomic dispersion has been widely documented in ethnobotanical systems where medicinal knowledge develops under ecological heterogeneity and limited access to formal veterinary services^24,31,37^.

Across broader African and South Asian contexts, the predominance of gastrointestinal disorders, parasitic infections, and dermatological conditions is a recurrent pattern in ethnoveterinary systems, reflecting shared livestock management constraints, environmental exposure, and disease ecology. Comparable findings have been reported in Ethiopia, Nepal, India, and West Africa^22,27,29,38^. However, the exceptionally high proportion of single-species families observed in this study exceeds that reported in many comparable investigations, suggesting a more diversified therapeutic repertoire. This may reflect agroecological heterogeneity, long-term experiential learning, and localized innovation in ethnoveterinary practice, as also noted in other Ethiopian landscapes^24,32^.

Herbaceous species were the most frequently used growth forms, likely due to their ecological abundance, accessibility, and rapid regeneration within mixed crop–livestock systems^31,32^. Leaves constituted the most commonly harvested plant part (46.5%), a pattern generally interpreted as relatively sustainable in ethnobotanical literature^11,32^. However, the notable use of roots (18.9%) and bark (10.7%), combined with strong dependence on wild habitats (66.7%), raises significant conservation concerns. Destructive harvesting practices are widely recognized as major drivers of medicinal plant decline because they directly reduce plant survival and regeneration capacity, particularly for woody and slow-growing species^24,32,37^. These findings underscore the need for community-based cultivation of priority species, sustainable harvesting guidelines, and in situ conservation of wild populations.

The predominance of gastrointestinal disorders, ecto-/endoparasitic infections, and dermatological diseases reflects the major livestock health challenges faced by smallholder farmers in Ethiopia and elsewhere^22,32,38^. While blackleg emerged as a highly cited specific disease due to its severity and economic impact, gastrointestinal disorders constituted the largest category of use reports because they encompass multiple conditions such as diarrhoea, bloating, and internal parasitism. This distinction highlights the importance of differentiating between individual disease frequency and broader syndromic categories in ethnoveterinary studies^22,32^.

Local disease diagnosis is largely symptom-based and grounded in experiential knowledge developed through long-term observation of livestock behaviour, disease progression, and treatment outcomes. This represents a structured indigenous diagnostic system that integrates empirical observation with cultural interpretation of disease causation^26,51^. The bilingual English–Afaan Oromo classification system applied in this study improved alignment between vernacular and biomedical terminology, reducing ambiguity in disease categorization and enhancing comparability with veterinary science frameworks^9,13,17^. Continued collaboration with veterinary professionals could further refine diagnostic equivalence and strengthen ethnoveterinary integration into formal animal health systems.

Ethnobotanical indices such as Informant Consensus Factor (ICF) and Fidelity Level (FL) are useful for quantifying knowledge distribution patterns but should be interpreted cautiously, as they do not directly measure pharmacological efficacy^53^. In this study, these indices were triangulated with qualitative interviews, preference ranking, direct matrix ranking, and market surveys to improve analytical robustness and reduce methodological bias^22,32,36^. Reproductive disorders showed the highest ICF value (0.67), followed by dermatological (0.58) and respiratory conditions (0.52), indicating relatively strong informant agreement on plant use for these categories. High FL values recorded for Embelia schimperi, Ocimum lamiifolium, and Phytolacca dodecandra further indicate strong ailment-specific cultural importance and consistent therapeutic application^18,19^. These quantitative patterns of agreement and specificity provide a foundation for interpreting preference-based assessments of perceived therapeutic efficacy.

Preference ranking, focused on diarrhea due to its representation of the largest ailment category (28.5% of use reports) and its economic significance in smallholder systems, identified Hagenia abyssinica as the most preferred medicinal plant for treating livestock diarrhoea, followed by Zingiber officinale and Embelia schimperi. This pattern likely reflects both perceived efficacy and the established pharmacological relevance of these species in Ethiopian traditional medicine, particularly for gastrointestinal and anthelmintic applications^8,10,15^. The strong agreement between preference ranking and fidelity level further suggests that repeated empirical validation within communities reinforces cultural preference for certain medicinal species. However, such rankings should be interpreted as indicators of perceived efficacy rather than confirmed pharmacological potency.

Direct matrix ranking further revealed that Hagenia abyssinica, Croton macrostachyus, Podocarpus falcatus, and Juniperus procera face the highest multipurpose use pressure due to their simultaneous utilization for medicine, firewood, construction, fencing, tools, and shade (Table 4). Species with high composite use values are particularly vulnerable to overharvesting because medicinal demand compounds pressure from other utilitarian needs. This finding reinforces the conservation priority of these taxa and supports targeted interventions such as community-based propagation, rotational harvesting protocols, and integration of these species into agroforestry systems to reduce extraction pressure on wild populations.

Ethnoveterinary knowledge in Aseko District is predominantly transmitted orally and concentrated among older generations, consistent with patterns reported in many rural African communities^14,15^. The higher number of species reported by older informants suggests progressive intergenerational erosion of knowledge, likely driven by formal education, livelihood transformation, and reduced reliance on traditional livestock healthcare systems. The relatively lower participation of women reflects sociocultural constraints on knowledge exchange rather than absence of knowledge, as documented in other ethnobiological studies in Ethiopia^14,32^. This highlights the need for more inclusive documentation strategies that capture gendered dimensions of ethnoveterinary knowledge.

Market-based observations indicate that several medicinal plants also possess economic value, contributing to household income while simultaneously increasing harvesting pressure on wild populations. Quantitative threat assessment (Table 5) identified agricultural expansion (22.0%) as the most significant pressure on medicinal plant resources, followed by firewood and charcoal production (19.8%), climate stress including drought and erosion (18.7%), construction and timber extraction (15.9%), grazing pressure (14.8%), and pesticide use (12.1%). These drivers reflect the interconnected nature of livelihood dependence and ecological degradation in rural Ethiopia. Similar threat patterns have been widely reported in ethnobotanical and ethnoveterinary studies across Africa and Asia^26,27,37^. Therefore, integrated conservation strategies that combine biodiversity protection with livelihood support are essential. Community-based approaches such as home-garden cultivation of priority species (e.g., Hagenia abyssinica, Ruta chalepensis), protection of culturally significant plant sites, and participatory harvesting regulations are more likely to ensure sustainability than externally imposed conservation measures.

Several limitations should be acknowledged. The purposive sampling approach enhanced the inclusion of knowledgeable informants but may limit statistical generalizability and influence consensus-based indices. Although extended fieldwork improved seasonal representation, data remain dependent on informant recall and perception. Furthermore, while voucher specimens were authenticated and deposited in the Ethiopian Biodiversity Institute National Herbarium^47^, ethnoveterinary evidence alone cannot confirm pharmacological efficacy. Therefore, species identified through high consensus, fidelity, and preference ranking should be prioritized for phytochemical, toxicological, and pharmacological validation^24,32,53^.

In conclusion, ethnoveterinary knowledge in Aseko District represents a biologically rich, culturally embedded, and functionally important system that is simultaneously resilient and vulnerable. While it supports primary livestock healthcare in resource-limited settings, it is increasingly threatened by ecological degradation and sociocultural transformation. Sustaining this system will require integrated strategies including conservation-oriented cultivation of high-use species, regulation of destructive harvesting practices, scientific validation of priority medicinal plants, inclusive documentation of indigenous knowledge, and long-term ecological monitoring. Collaborative engagement among local communities, veterinary professionals, conservation practitioners, and researchers is essential to ensure the sustainable coexistence of livestock health needs, biodiversity conservation, and cultural heritage^22,24,26,32,36,38,40,53^.

## Materials and methods

### Study area

**Fig. 5:**
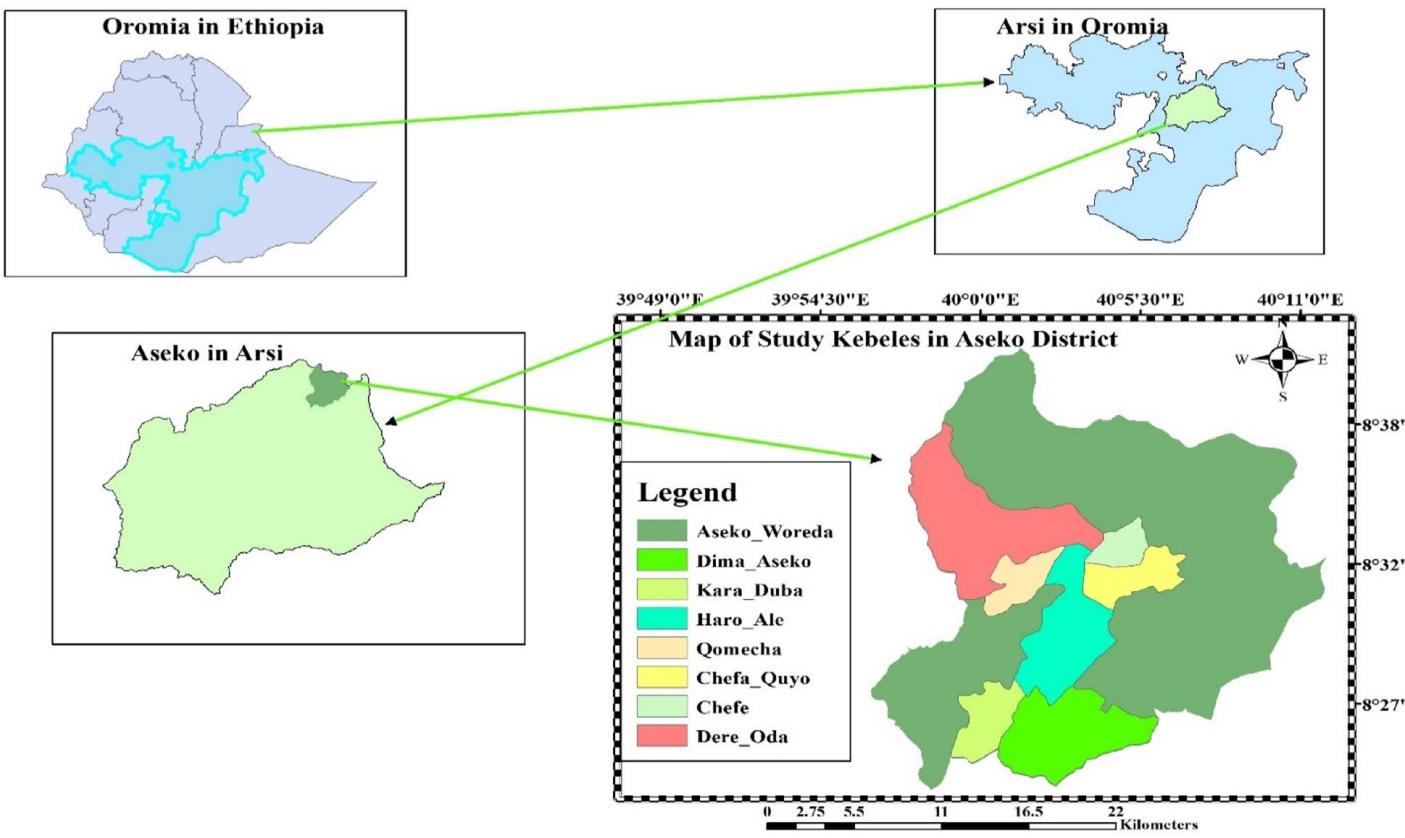
Location map of Aseko and study sites. The map shows the geographical position of Aseko District within the Arsi Zone of Oromia Regional State, southeastern Ethiopia, and the locations of the seven kebeles surveyed.

The study was conducted in Aseko District, Arsi Zone, Oromia Regional State, southeastern Ethiopia (Fig. 5). The district lies between 8°15′10″–8°41′00″ N latitude and 39°55′40″–40°16′20″ E longitude, approximately 160 km southeast of Addis Ababa^34^. It covers an area of 600–610 km² with elevations ranging from 1,170 to 2,950 m above sea level^34^. Mean annual rainfall ranges from 600 to 1,200 mm, and mean annual temperature varies from 12 to 24 °C across the agroecological gradient.

The district comprises three agroecological zones: highland (> 2,500 m), midland (1,800–2,500 m), and lowland (< 1,800 m). These zones differ in climate, vegetation structure, and land-use patterns, which strongly influence medicinal plant availability and ethnoveterinary practices. Vegetation types include dry evergreen Afromontane forest, shrubland, and grassland ecosystems that support high plant diversity^34^.

### Reconnaissance survey and site selection

A reconnaissance survey was conducted in June 2024 to assess vegetation distribution, settlement patterns, livestock systems, accessibility, and ethnoveterinary knowledge availability. Based on consultations with agricultural officers, development agents, veterinary professionals, and local elders, seven kebeles were purposively selected across the three agroecological zones.

Selection criteria included: (i) presence of experienced livestock keepers or traditional healers, (ii) accessibility for repeated field visits, and (iii) representation of distinct vegetation and land-use systems. Ethical clearance and specimen collection permit (EBI/RES/057/2024) were obtained prior to fieldwork. Preliminary administrative coordination with local authorities was conducted before field implementation to ensure smooth ethical approval and logistics.

### Informant selection

Informants were selected using purposive and snowball sampling methods commonly applied in ethnobotanical studies^49,50^.

Thirty key informants were selected based on: (i) ≥ 10 years ethnoveterinary experience, (ii) recognition by community leaders as practitioners, and (iii) active involvement in livestock healthcare. Informants not actively practicing ethnoveterinary medicine in the past five years were excluded.

In addition, 152 household respondents were selected proportionally across the seven kebeles. Sample size was determined using the Krejcie and Morgan formula^48^:

$${\mathrm{n}} = ({\mathrm{NZ}}^{{\mathrm{2}}} {\mathrm{p}}({\mathrm{1}} - {\mathrm{p}}))/({\mathrm{d}}^{{\mathrm{2}}} ({\mathrm{N}} - {\mathrm{1}}) + {\mathrm{Z}}^{{\mathrm{2}}} {\mathrm{p}}({\mathrm{1}} - {\mathrm{p}}))$$

where *N* = 8,665 households, Z = 1.96, *p* = 0.80, and d = 0.08. The calculated minimum sample size was 95, but this was increased to 152 to improve representativeness across agroecological zones and reduce sampling error.

### Data collection methods

Data were collected between July 2024 and February 2025 using semi-structured interviews, focus group discussions (FGDs), guided field walks, and market surveys following standard ethnobotanical protocols^40,50^.

Semi-structured interviews (45–90 min) were conducted in Afaan Oromo and recorded using field notes with consent. Data included plant species, parts used, preparation methods, dosage, routes of administration, and livestock ailments treated.

FGDs (6–8 participants each) were conducted in all seven kebeles to triangulate interview data and validate ethnoveterinary knowledge^53^.

Guided field walks enabled in situ identification and documentation of medicinal plants in farmland, grazing areas, forest patches, and home gardens^34,53^.

All ethnoveterinary use reports are compiled in Supplementary Table S1, including plant species, local names, ailments, preparation methods, routes of administration, voucher numbers, and informant citations (151 use reports from 66 species).

### Market survey methodology

Market surveys were conducted in Aseko, Dima, and Irreessa markets between September 2024 and January 2025. A total of 27 medicinal plant vendors were interviewed.

Data included plant availability, trade names, plant parts sold, demand, and price. Prices were recorded using local measurement units (bundles, cups, spoons, bottles, and sacks). Each market was visited at least three times to ensure data reliability and consistency.

### Disease classification validation

Livestock diseases were initially recorded using Afaan Oromo local names. These were translated into standard veterinary terminology through consultation with district veterinary officers and animal health professionals.

Diagnostic equivalence was validated based on symptoms, affected species, disease progression, and treatment response, with reference to standard veterinary literature^4,6^. This process ensured consistency between indigenous and biomedical disease classification systems.

### Voucher specimen collection and identification

Medicinal plant specimens were collected during field walks, pressed, dried, labeled, and processed following herbarium standards^43^.

Identification was performed using the Flora of Ethiopia and Eritrea^1^ and confirmed at the Ethiopian Biodiversity Institute (EBI) National Herbarium in collaboration with taxonomists from Wondo Genet College of Forestry and Natural Resources, Hawassa University.

Voucher specimens were deposited at the EBI National Herbarium. Voucher numbers (JSJ-001–060) are provided in Supplementary Table S1 following Index Herbariorum standards^52^.

### Ethical considerations

Ethical clearance was obtained from the Aseko District Administration and Aseko District Agricultural and Natural Resources Office. The study followed the International Society of Ethnobiology (ISE) Code of Ethics^45^.

Verbal informed consent was obtained from all participants after explaining the study objectives in Afaan Oromo. Participation was voluntary, and no personal identifiers are included.

Permits for plant collection were obtained from EBI (Permit No. EBI/RES/057/2024). No endangered or CITES-listed species were harvested. Sampling was limited to minimal quantities required for identification and voucher preparation.

### Quantitative ethnobotanical analysis

Data were analyzed using descriptive statistics (frequencies and percentages) and ethnobotanical indices. Analyses were performed using IBM SPSS Statistics v26 and Microsoft Excel 2019. *“Growth form (herb*,* shrub*,* tree*,* climber) and habitat source (wild*,* cultivated) were recorded for each species to assess harvesting pressure and conservation vulnerability.*

Ailment categories with fewer than two use reports were excluded from index calculations to ensure statistical robustness^53^.

### Informant Consensus Factor (ICF)

ICF was calculated following Trotter and Logan^53^:

$${\mathrm{ICF}} = ({\mathrm{Nur}} - {\mathrm{Nt}})/({\mathrm{Nur}} - {\mathrm{1}})$$

where Nur = number of use reports and Nt = number of taxa. Values closer to 1 indicate higher consensus.

### Fidelity Level (FL)

FL was calculated as^19^:

$${\mathrm{FL}}\left( \% \right) = \left( {{\mathrm{Ip}}/{\mathrm{Iu}}} \right) \times {\mathrm{1}}00$$

where Ip = number of informants citing a species for a specific ailment and Iu = total informants (*n* = 182) reporting that species.

### Preference ranking

Preference ranking was conducted**Ranking of Medicinal Plants** with 14 key informants using seven antidiarrheal species scored from 1 (least effective) to 7 (most effective)^54^.

Diarrhea was selected due to: (i) high frequency (28.5%, Table 2), (ii) major economic impact, and (iii) established ethnoveterinary relevance of key species such as *Hagenia abyssinica*. Scores were summed across informants.

Preference ranking was conducted to assess the perceived effectiveness of medicinal plants used for treating the most frequently reported livestock ailment in the study area. Livestock diarrhoea was selected for this exercise because it was the most commonly cited and economically significant disease across all surveyed kebeles, making it suitable for comparative evaluation of antidiarrheal remedies.

Fourteen purposively selected key informants, identified based on their extensive ethnoveterinary knowledge, participated in the ranking exercise. Seven commonly reported medicinal plant species used for treating diarrhoea were evaluated. Each informant independently assigned scores ranging from 1 (least effective) to 7 (most effective) based on their perceived therapeutic efficacy derived from personal experience and community knowledge.

The scores assigned by all informants were summed for each species, and the total values were used to determine the final rank, with higher scores indicating greater perceived effectiveness.

### Direct matrix ranking (DMR)

DMR was conducted with 12 key informants scoring eight species across eight use categories (0–5 scale).

Composite values were calculated as mean scores across informants and use categories. Higher scores indicate greater multipurpose pressure and conservation priority.

### Threat assessment

Seven key informants scored six threat factors (1–7 scale). Scores were summed and converted into percentages.

Threats assessed included agricultural expansion, fuelwood collection, climate stress, construction pressure, grazing, and pesticide use^24,37^. *Direct matrix ranking was used to evaluate multipurpose use pressure on high-conservation-priority species identified through informant consensus and ecological vulnerability.*

### Ranking of threat factors

A ranking exercise was conducted to assess the relative importance of major threats to medicinal plants in the study area. Seven key informants (R1–R7) were selected based on their knowledge of local environmental conditions. Each informant assigned scores to identified threat factors based on their perceived severity.

Scores were summed across informants to determine total values, which were then converted into percentages and ranked accordingly.

### Statistical analysis

Descriptive statistics were used for demographic and ethnobotanical patterns. Ethnobotanical indices (ICF, FL) and ranking outputs were calculated as described above.

No inferential tests were applied due to purposive sampling design; results are interpreted descriptively. All outputs are presented with appropriate summary statistics.

## Supplementary Information

Below is the link to the electronic supplementary material.

Supplementary Material 1

## Data Availability

All data analyzed during this study are included in this published article and its Supplementary Information files. Ethnoveterinary use reports, species lists, and quantitative indices are provided in **Supplementary Table S1** . Voucher specimen accession numbers are available from the corresponding author upon reasonable request.

## Declarations

### Approval of animal experiments

Not applicable. This study did not involve experiments on live vertebrate or invertebrate animals.
