# Supplementary material for: Ethnoveterinary medicinal plants in Aseko District, Arsi Zone, southeastern Ethiopia
Source: Sci Rep. 2026 May 29;16:24639. doi: 10.1038/s41598-026-53207-x (PMC13454274; doi:10.1038/s41598-026-53207-x)
Supplement: Supplementary file 1 [file 41598_2026_53207_MOESM1_ESM.pdf]

Study of ethnoveterinary medicinal plants in Aseko District, Arsi Zone, southeastern Ethiopia

Authors
Jewar Safeno Jara[1]*, Melesse Maryo[2], Zerihun Girma[3]

Affiliations
[1]Department of General Forestry, Mizan Tepi University, P.O. Box 260, Mizan Aman, Ethiopia
[2]Ethiopian Biodiversity Institute; home-based at Ethiopian Civil Service University, Addis Ababa, Ethiopia
[3]Wondo Genet College of Forestry and Natural Resources, Hawassa University, Shashemene, Ethiopia

Corresponding author: Jewar Safeno Jara[1]*
Email: jewar04@gmail.com; jewarsafeno@mtu.edu.et
ORCID: https://orcid.org/0000-0001-9879-6315

Supplementary Table S1 | **Complete ethnoveterinary use reports from Aseko District, southeastern Ethiopia**

This dataset comprises **151 individual use reports** collected from **182 informants** across seven kebeles in Aseko District. It includes:

- Scientific name and family of each medicinal plant (66 species total)
- Local name in Afaan Oromoo
- Livestock ailment treated (33 ailments)
- Plant part(s) used
- Preparation method (e.g., crushing, decoction, pounding)
- Route of administration (oral, dermal, nasal, etc.)

All data were recorded during semi-structured interviews and guided field walks between July 2024 and February 2025. Voucher specimens were deposited at the Ethiopian Biodiversity Institute Herbarium (available upon request).

Abbreviations:

PU: Plant parts used — Bark (Ba), Latex (La), Root (R), Leaf (L), Fruit (Fu), Flower (F), Seed (Se), Stem (St), Bulb (Bu), Rhizome (Rh), Twigs (Tw), Whole plant (Wp), Root+Leaf (RL), Leaf+Bark (LBa), Sap/Latex (Sa) RA: Route of administration — Oral (O), Nasal (N), Ocular (Op), Dermal (Dm), Auricular (Au) CO: Condition — Fresh (F), Dried (D), Fresh or dried (F/D) Ha: Growth habit — Herb (H), Shrub (Sh), Tree (T), Climber (Cl)

Ct: Collection source — Wild (W), Home garden (Hg)

Voucher ID	Scientific name	Family	Local name	PU	Disease treated	Mode of preparation with dosage used	RA	CO	Ha	Ct
EBI-2024-0678	Achyranthes aspera L.	Amaranthaceae	Darguu	L	Eye problem	Direct application: fresh leaves chewed and the juice applied directly to the eye	Op	F	H	W
EBI-2024-0679	Aeonium leucoblepharum Webb ex A. Richard	Crassulaceae	Harmaa gurraatti	L	Retained placenta	Pounding and drenching: fresh leaves pounded, mixed with cold water, and given orally using a local cup (Merti)	O	F	H	W
EBI-2024-0680	Agave sisalana	Agavaceae	Algee	R	Blackleg	Crushing and drenching: fresh root crushed, mixed with water, and given orally to cattle	O	F	H	W
EBI-2024-0681	Aloe vera L.	Aloaceae	Hargiisa	L	Bloating	Pounding and drenching: leaves pounded with Ruta chalepensis, Allium sativum, and Foeniculum vulgare, mixed with water, and given orally using a large local container	O	F	H	W
EBI-2024-0682	Allium sativum L.	Alliaceae	Qullubii-Adii	Bu	Pasteurellosis	Pounding and drenching: bulbs pounded with roots of Brucea antidysenterica, Croton macrostachyus, and Premna resinosa, mixed with water, and poured onto infected area	Dm	F	H	Hg
				Bu	Bloating	Pounding and drenching: ten bulbs pounded and given orally twice daily for 3–5 days	O	F		
				Bu	Blackleg	Crushing and drenching: bulbs crushed, mixed with cold water, and poured onto infected part	Dm	F/D		
				Bu	Dermatophilosis	Direct application: bulbs ground and powder applied to affected skin	Dm	F/D		
				Bu	Scabies	Direct application: bulbs ground and powder applied to affected skin	Dm	F/D		
				Bu	Ringworm	Direct application: bulbs ground and powder applied to affected skin	Dm	F/D		
				Bu	Parasitic leech	Crushing and drenching: bulbs pounded, mixed with cold water, and given orally or poured onto affected area	O / Dm	F/D		

				Bu	Lice infestation in chicken	Direct application: juice extracted and painted onto affected body parts	Dm	F		
EBI-2024-0683	Anethum graveolens L.	Apiaceae	Koomana	R	Retained feces	Concoction: roots pounded, mixed with water, and the preparation drunk orally	O	F	H	Hg
EBI-2024-0684	Asparagus africanus Lam.	Asparagaceae	Sariitii	L	Placenta retention	Crushing and drenching: leaves and roots pounded, mixed with cold water (without boiling), and given orally using a large local cup (Merti)	O	F	H	W
				R	Coccidiosis	Decoction: roots boiled, cooled, and the liquid given orally	O	F		
				R	Wound	Crushing, pasting, and tying: fresh roots pounded with leaves of Dodonaea angustifolia, mixed with butter, and applied to the wound	Dm	F		
EBI-2024-0685	Brassica nigra (L.) Koch	Brassicaceae	Siinaficaa	Se	Bloating	Decoction: seeds boiled with salt, cooled, and drunk orally	O	D	H	Hg
				Se	Bloating	Concoction: seeds powdered and taken orally with water	O	D		
EBI-2024-0686	Brucea antidysenterica J.F.Mill.	Simaroubaceae	Jiloolaafaa	R	Wound	Crushing, pasting, and tying: root crushed with fresh leaves of Dodonaea angustifolia and tied on the wound every other day for two days	Dm	F/D	Sh	W
EBI-2024-0687	Calpurnia aurea (Ait.) Benth.	Fabaceae	Cekaa	L	Ectoparasites	Direct application: crushed plant material washed and applied to affected areas	Dm	F	Sh	W
				RL	Mechanical trauma	Direct application: leaves and roots smashed and rubbed onto affected area	Dm	F		
				L	Tick infestation	Direct application: juice extracted and painted onto affected parts	Dm	F		
				L	Helminthiasis	Crushing and drenching: leaves pounded, mixed with cold water, and given orally	O	F		
				L	Snake bite	Crushing and drenching: plant material crushed, mixed with water, and forcefully administered orally	O	F		

				R	Snake bite	Crushing and drenching: roots pounded, mixed with cold water, and given orally	O	F		
				L	Sore	Direct application: plant parts ground and pasted onto affected skin	Dm	F/D		
				Bu	Parasitic leech	Crushing and drenching: plant material pounded, mixed with water, and given orally or poured onto affected area	N	F/D		
EBI-2024-0688	Capsicum annuum L.	Solanaceae	Barberee	Fu	Bloat	Pounding and drenching: fruits pounded, mixed with water, and given orally	O	D	H	Hg
EBI-2024-0689	Carissa spinarum L.	Apocynaceae	Hagamsa	R	Evil eye	Direct application: root powdered, mixed with butter, and creamed onto affected body part	Dm	D	Sh	W
				R	Helminthiasis	Crushing and drenching: juice extracted and given orally	O	F		
EBI-2024-0690	Capparis tomentosa Lam.	Capparidaceae	Harangamaa	R	Breast swelling	Crushing, pasting, and tying: fresh root pounded, mixed with butter, and applied to the swollen breast	Dm	F/D	Sh	W
EBI-2024-0691	Catha edulis (Vahl) Forssk. ex Endl.	Celastraceae	Jimaa	L	Urine retention	Pounding and drenching: leaves pounded with Ruta chalepensis and Foeniculum vulgare, mixed with water and local areke (katikala), and given orally	O	F	Sh	Hg
EBI-2024-0692	Cissampelos mucronata A. Rich.	Menispermaceae	Bishaan qal'ee	L	Respiratory infection	Crushing and drenching: leaves crushed with Nicotiana tabacum, mixed with honey, and administered orally or through nostrils	O / N	F	Cl	W
EBI-2024-0693	Clutia abyssinica Jaub. & Spach	Euphorbiaceae	Ulee foonii	L	Bloody diarrhoea	Crushing and drenching: leaves pounded, mixed with cold water, and given orally	O	F	Sh	Hg
				R	Nasal infection	Crushing and drenching: roots pounded, mixed with cold water, and administered nasally	N	F		
				R	Mucous diarrhoea	Crushing and drenching: roots pounded, mixed with cold water, and given orally	O	F		

				L	Bloat	Crushing and drenching: leaves pounded, mixed with cold water, and given orally	O	F		
				L	Ophthalmic infection	Direct application: juice extracted and applied directly to the eye	Op	F		
EBI-2024-0694	Croton macrostachyus Del.	Euphorbiaceae	Makanisaa	L	Bloating	Crushing and drenching: leaves crushed and administered nasally	N	F	T	W
				L	Stomachache	Crushing and drenching: sap squeezed and drunk orally	O	F		
				La	Wound	Direct application: harvested latex painted onto wound	Dm	F		
				R	Rabies	Concoction: dried root powdered and mixed with injera, given to dogs	O	F/D		
				L	Ringworm	Direct application: juice extracted and painted onto affected area	Dm	F		
				L	Dermatophilosis	Direct application: juice extracted and painted onto affected area	Dm	F		
				L	Minor bleeding	Direct application: plant parts ground and pasted onto affected area	Dm	F		
				L	Sore	Direct application: plant parts ground and pasted onto affected area	Dm	F/D		
				L	Scabies	Direct application: juice extracted and painted onto affected skin	Dm	F		
EBI-2024-0695	Cucumis ficifolius A. Rich.	Cucurbitaceae	Araddoo gurraatti	R	Bloating and blackleg	Crushing and drenching: roots crushed with Vernonia amygdalina, Allium sativum, Ruta chalepensis, and leaves of Justicia schimperiana, mixed with water and given orally to cattle	O	F	Cl	W
				R	Pasteurellosis	Pounding and drenching: roots pounded with Croton macrostachyus, Allium sativum, Premna schimperi, Brucea antidysenterica, and Maesa lanceolata, mixed with water and given orally	O	F		
				L	Dislocated leg	Direct application: fresh leaves mixed with butter, warmed on fire, and applied to affected part	Dm	F		

				R	Cattle infection	Pounding and drenching: roots pounded with leaves of Teclea nobilis, mixed with cold water, and two glasses given orally to cattle	O	F/D		
EBI-2024-0696	Cucurbita pepo L.	Cucurbitaceae	Dubbaa / Dhobba	R	Bloat	Pounding and drenching: roots pounded with Vernonia amygdalina, mixed with local areke (katikala), and given orally or sprinkled on feed	O	F	H	Hg
				LBa	Rabies	Concoction: leaves and bark pounded; ½ coffee cup given to humans and 1 cup to cattle	O	F		
EBI-2024-0697	Cynodon dactylon (L.) Pers.	Poaceae	Coqorsa	Wp	Snake bite	Crushing, pasting, and tying: entire above-ground parts crushed, mixed with butter, and rubbed onto snake-bitten area	Dm	F	H	W
EBI-2024-0698	Dodonaea angustifolia L.f.	Sapindaceae	Iticha	L	Ear wound	Crushing, pasting, and tying: leaves crushed with butter and placed on affected ear	Au	D	Sh	W
				Se	Internal parasites	Concoction: plant material ground, mixed with oat flour, baked, and fed to animals	O	F/D		
				L	Wound	Direct application: fresh or dried leaves ground and applied after washing wound with squeezed leaves of Calpurnia aurea	Dm	F		
EBI-2024-0699	Discopodium eremanthum Chiov.	Solanaceae	Mararo	L	Abdominal distension	Concoction: leaves crushed and chewed or drunk as liquid preparation	O	F	Sh	W
				L	Retained placenta	Decoction: fresh leaves pounded and the boiled liquid drunk	O	F		
				L	Blackleg	Concoction: leaves crushed and the preparation drunk orally	O	F/D		
				L	Breast pain	Direct application: crushed leaves used as steam bath	Dm	F/D		
EBI-2024-0700	Dovyalis abyssinica A. Rich.	Flacourtiaceae	Kooshimo	L	Retained placenta	Decoction: leaves pounded and boiled; liquid drunk orally	O	F	Sh	W
EBI-2024-0701	Embelia schimperi Vatke	Primulaceae	Gurraa / Qorqoro	R	Gastrointestinal worms	Decoction: dried roots pounded, boiled in water for 15 minutes; one coffee cup	O	D	Sh	W

						given orally to cattle twice daily for 3 days				
				R	Diarrhea	Same decoction administered once daily until symptoms subside	O	D		
EBI-2024-0702	Erythrina brucei Schweinf.	Fabaceae	Walleensu	L	Eye problem	Direct application: sap squeezed and applied directly to the eye	Op	F	T	W
				L	Cough	Concoction: crushed leaves mixed with food and given to hens	O	F		
				Ba	Wound	Direct application: powdered bark applied to wound	Dm	D		
EBI-2024-0703	Eucalyptus globulus Labill.	Myrtaceae	Bargamo adii	L	Cholera	Decoction: leaves pounded, boiled, liquid mixed with wheat flour and fed to hens	O	F	T	Hg
EBI-2024-0704	Euphorbia abyssinica J.F. Gmel.	Euphorbiaceae	Adaami	L	Mule malaria	Direct application / smoke inhalation: fumigation using leaves	N	F	T	W
				L	Rinderpest	Direct application / smoke inhalation: fumigation of animal	N	F		
				Ba	Ascaris	Concoction: powdered bark of Croton macrostachyus mixed with food and taken orally	O	F		
EBI-2024-0705	Hagenia abyssinica (Bruce) J.F. Gmel.	Rosaceae	Heexoo	L	Retained placenta	Direct consumption: fresh leaves harvested and fed directly to cattle	O	F	T	W
EBI-2024-0706	Heteromorpha arborescens (Spreng.) Cham. & Schltdl.	Apiaceae	Aliyaanqaa	R	Eye disease	Direct application: fine root powder mixed with minimal water and applied to eye	Op	F/D	Sh	W
EBI-2024-0707	Hordeum vulgare L.	Poaceae	Garbuu	Se	Bloat	Concoction: seeds crushed with dry leaves of Melia azedarach and sprinkled on feed	Dm	F/D	H	Hg
				Se	Broken bones and worn tissues	Concoction: seeds lightly toasted, ground, prepared as soup for humans; grains fed daily to sick equines	O	D		
EBI-2024-0708	Hypericum revolutum Vahl	Hypericaceae	Garambaa	LR	Rabies	Pounding and drenching: roots and leaves pounded with Rumex nervosus, Phytolacca dodecandra, Brucea antidysenterica, and Croton	O	F/D	Sh	W

						macrostachyus, mixed with water and given orally				
EBI-2024-0709	Juniperus procera L.	Cupressaceae	Hindheessa	R	Retained placenta / fatal membrane	Crushing and drenching: root pounded, mixed with cold water, and drunk or poured on animal	O	F	T	W
EBI-2024-0710	Justicia schimperiana (Hochst. ex Nees) T. Anders.	Acanthaceae	Dhumugaa /Sensel	LR	Rabies	Crushing and drenching: plant pounded with cold water and given orally or poured on animal	O	F	Sh	W
				LR	Blackleg	Crushing and drenching: same preparation, administered orally	O	F/D		
				L	Intestinal parasites	Crushing and drenching: same preparation, administered orally	O	F/D		
EBI-2024-0711	Lepidium sativum L.	Brassicaceae	Fexo / Shuunfaa	Se	Bloating	Pounding and drenching: seeds pounded with bulb of Allium sativum and given orally	O	D	H	Hg
				Se	Febrile illness	Direct application / smoke inhalation: seeds placed on fire and smoke inhaled	N	D		
EBI-2024-0712	Linum usitatissimum L.	Linaceae	Talbaa	Se	Retained placenta	Concoction: seed powder dissolved in water; half glass given orally	O	D	H	Hg
				Se	Constipation	Infusion: seeds soaked overnight and solution drunk	O	D		
EBI-2024-0713	Maesa lanceolata Forssk.	Myrsinaceae	Abbayyii	L	Ectoparasites	Direct application: plant material pounded and painted onto skin	Dm	F	T	W
				R	Pasteurellosis (Gorosisu)	Concoction: roots pounded with Allium sativum, Brucea antidysenterica, Croton macrostachyus, and Cucumis ficifolius, mixed with porcupine meat; 1 bottle for adult cattle, ½ for calves	O	F/D		
				L	Tick infestation	Direct application: juice extracted and painted on affected parts	Dm	F		
				Fu	Dermatophilosis	Direct application: plant powder applied to skin	Dm	F		
				Fu	Helminthiasis	Crushing and drenching: plant pounded, mixed with water, and given orally	O	F		
				Fu	Parasitic leech	Crushing and drenching: same preparation administered orally	O	F		

EBI-2024-0714	Melia azedarach L.	Meliaceae	Mim	Ba	Anthrax	Concoction: dried bark powder mixed with water and drunk once	O	D	T	Hg
EBI-2024-0715	Musa × paradisiaca L.	Musaceae	Muuzii	Sa	Wound	Direct application: latex/sap applied directly with added salt	Dm	F	H	Hg
				Sa	Snake bite	Direct consumption: sap squeezed and drunk	O	F		
EBI-2024-0716	Myrsine africana L.	Myrsinaceae	Qacama	R	Rabies	Decoction: roots boiled and liquid drunk after cooling	O	F	Sh	W
EBI-2024-0717	Nigella sativa L.	Ranunculaceae	Habsuda guracha	Se	Blackleg	Decoction: seeds powdered and the decoction drunk	O	F/D	H	Hg
EBI-2024-0718	Nicotiana tabacum L.	Solanaceae	Timbaho / Tamboo	L	Cough	Crushing and drenching: sap squeezed and ½ litre given orally to cattle	O	F	H	Hg
				L	Leeches	Crushing and drenching: sap squeezed and 1 coffee cup given orally	O	F		
				LR	Bloating	Concoction: dried leaves and roots powdered, mixed with salt, and given orally	O	D		
				L	Snake bite	Crushing, pasting, and tying: leaves pounded and tied onto affected area	Dm	F		
EBI-2024-0719	Ocimum lamiifolium Hochst.	Lamiaceae	Damakasee	L	Diarrhea	Infusion: leaves boiled briefly, cooled, and 1 glass given orally twice daily	O	F	H	Hg
				L	Bloating	Leaves crushed with Allium sativum, mixed with water, and given orally	O	F		
EBI-2024-0720	Olinia rochetiana A. Juss.	Oliniaceae	Gunaa	L	Eczema	Crushing, pasting, and tying: crushed leaves tied onto affected skin	Dm	F	T	W
EBI-2024-0721	Phoenix reclinata Jacq.	Arecaceae	Meexxi	L, St	Eye disease	Direct application: leaves and stems chewed with Premna resinosa and spat into cattle's eye	Op	F	T	W
EBI-2024-0722	Phytolacca dodecandra L'Hér.	Phytolaccaceae	Handoodee	Ba	Rabies	Concoction: dried root powdered, soaked overnight; ½ coffee cup for adults, ¼ for children, 1 cup for cattle	O	D	Cl	W
				LR	Hyena bite	Crushing, pasting, and tying: root and leaf smashed and tied on neck with clean cloth	N	F		
				L	Parasitic leech	Crushing and drenching: plant pounded and administered orally	O	F		

				R	Mange	Crushing and drenching: same preparation administered orally	O	F		
				R	Helminthiasis	Crushing and drenching: same preparation administered orally	O	F		
				L	Lice infestation in chicken	Crushing and drenching: preparation given orally or poured on animal	O	F		
				R	Lumpy skin disease	Crushing and drenching: preparation administered orally	O	F		
EBI-2024-0723	Plantago lanceolata L.	Plantaginaceae	—	R	Rabies	Crushing and drenching: plant pounded with cold water and given orally or poured on animal	O	F	H	Hg
EBI-2024-0724	Podocarpus falcatus (Thunb.) Mirb.	Podocarpaceae	Birbirsa	Ba	Ringworm	Direct application: plant powder applied to affected skin	Dm	F	T	W
				Ba	Mange	Direct application: plant powder applied to affected skin	Dm	F		
EBI-2024-0725	Plumbago zeylanica L.	Plumbaginaceae	Merxes	L	Cough with mucus	Concoction: leaf preparation drunk once daily for three mornings	O	D	H	W
				R	Eye disease	Direct application: fine root powder mixed with drops of water and applied to eye	Op	D		
EBI-2024-0726	Premna schimperi Engl.	Lamiaceae	Urgessa	L	Eye disease	Direct application: leaves crushed with Ocimum forskolei, Vernonia amygdalina, Hagenia abyssinica, and Cynodon dactylon; drops applied to eye	Op	F	T	W
				R	Pasteurellosis	Concoction: roots pounded with Allium sativum, Brucea antidysenterica, and Cucumis ficifolius, mixed with porcupine meat; 1 bottle for adult cattle, ½ for calves	O	F/D		
EBI-2024-0727	Rhamnus prinoides L'Hér.	Rhamnaceae	Geeshoo	L	Leech	Crushing and drenching: fresh leaves pounded with Nicotiana tabacum and pepper, mixed with water and milk, and applied through the nostrils	N	F	Sh	Hg
EBI-2024-0728	Ricinus communis L.	Euphorbiaceae	Qobboo	R	Stomachache	Concoction: root powdered and mixed with leaves of Momordica foetida; ½ L of the preparation given orally	O	D	Sh	Hg

				R	Blackleg	Crushing and drenching: root crushed and powdered, mixed with soap; ½ L given orally	O	F		
				L	Bloating	Direct application / smoke inhalation: leaves crushed with Croton macrostachyus and sniffed	N	F		
				Fu	Anthrax	Concoction: dried fruits powdered, mixed with water; one tea cup given orally to cattle	O	D		
				R	Sudden sickness	Pounding and drenching: roots pounded with roots of Justicia schimperiana, mixed with cold water; 1–2 tea cups given orally	O	F		
				R	Actinomycosis (Kurufsisa)	Concoction: root pounded with table salt and soil; one glass given to cattle and half glass to goats and sheep	O	F/D		
				Fu	Ulcerative lymphangitis	Direct application: dried fruit powdered and mixed with bark powder of Prunus africana, creamed onto ulcerated skin of donkey	Dm	D		
				Fu	Epizootic lymphangitis	Direct application: dried fruit powdered and mixed with bark powder of Prunus africana, creamed onto ulcerated skin of horse and mule	Dm	D		
				L	Swelling	Direct application: leaves warmed on fire and rubbed onto swollen area	Dm	F		
EBI-2024-0729	Rubus steudnerii Schweinf.	Rosaceae	Dhukkoo	L	Diarrhea	Infusion: fresh leaves steeped in hot water for 10 minutes; liquid cooled and given orally (½ bottle for calves, 1 bottle for adult cattle)	O	F	Sh	W
EBI-2024-0730	Rumex nepalensis Spreng.	Polygonaceae	Shaabbee	R	Internal parasites	Infusion / Decoction: root chewed and swallowed, or boiled in water; one glass drunk once	O	D	H	W
EBI-2024-0731	Ruta chalepensis L.	Rutaceae	Teenaadaama	LBa	Coccidiosis (Bilii)	Pounding and drenching: bark and leaves pounded with root of Justicia schimperiana and given to hens with injera	O	F	H	Hg

				L	Stomachache	Pounding and drenching: fresh leaves pounded with garlic, mixed with one glass of milk and drunk	O	F		
EBI-2024-0732	Solanum anguivi L.	Solanaceae	Hidi-bude	Se	Ticks	Direct application: crushed seeds applied directly to affected area	Dm	F	H	W
EBI-2024-0733	Sorghum bicolor (L.) Moench	Poaceae	Bisingaa caabbi	Se	Retained placenta	Concoction: dry seeds mixed with salt and water and given orally to cattle	O	D	H	Hg
				R	Rabies	Concoction: dry root powdered, baked with teff flour, and given orally to cattle	O	F		
				RL	Pasteurellosis	Concoction: dry roots and leaves of Stephania abyssinica powdered, mixed with water, and given orally to animals	O	D		
				L	Eye disease	Direct application: leaves crushed with Acacia abyssinica and Cynodon dactylon; drops applied to eye	Op	F		
EBI-2024-0734	Syzygium guineense var.	Myrtaceae	Baddessa	Ba	Leech infection	Direct application / smoke inhalation: inner stem bark chopped, prepared as solution, and half coffee cup administered through nostril	N	F	T	W
EBI-2024-0735	Teclea nobilis Del.	Rutaceae	Hadheesa	L	Trypanosomiasis (Trip)	Pounding and drenching: leaves pounded, mixed with water, and given orally to animals	O	F	Sh	W
				R	Wound	Direct application: root pounded, mixed with cold water, and applied to wound of donkey	Dm	F/D		
EBI-2024-0736	Thymus schimperi Ronniger	Lamiaceae	Xooshine	L	Gara Bokoyso	Concoction: leaves crushed and chewed or drunk as preparation	O	D	H	W
EBI-2024-0737	Tragia cinerea (Pax)	Euphorbiaceae	Laalessaa	L	Blackleg	Crushing and drenching: leaves pounded, solution prepared; 1 L given orally and small amount through nostrils	O/N	F	Cl	W
EBI-2024-0738	Trichocladus ellipticus Eckl. & Zeyh.	Hamamelidaceae	Dhadhaa	L	Eye disease	Direct application: leaf sap extracted and 2–3 drops applied directly to the eye twice daily	Op	F	Sh	W
EBI-2024-0739	Urera hypselodendron (A. Rich.) Wedd.	Urticaceae	Haliilaa	Wp	Urine retention	Pounding and drenching: plant pounded, mixed with water, and given orally	O	F	Cl	W

EBI-2024-0740	Verbascum sinaiticum Benth.	Scrophulariaceae	Gurra Harree	L	Diarrhoea	Concoction: leaf powder; 2–3 spoons with one bottle of water for cattle, one spoon with one cup for humans	O	F/D	H	W
EBI-2024-0741	Vernonia amygdalina Del.	Asteraceae	Ebicha	L	Diarrhoea	Concoction: leaf powder mixed with salt and given orally to cattle	O	F	Sh	W
				L	Wound	Direct application: sap squeezed and painted on wound	Dm	F		
EBI-2024-0742	Withania somnifera (L.) Dunal	Solanaceae	Gizaawa / Uunjoo	R	Blackleg	Crushing and drenching: root pounded with cold water and given orally or poured on affected part	O	F/D	Sh	W
EBI-2024-0743	Zingiber officinale Roscoe	Zingiberaceae	Zinjibila	Rh	Bloat	Crushing and drenching: rhizome pounded with cold water and given orally or poured on animal	O	F	H	Hg
Voucher ID				Rh	Bloody diarrhoea	Crushing and drenching: same preparation administered orally	O	F		
EBI-2024-0678				Rh	Mucous diarrhoea	Crushing and drenching: same preparation administered orally	O	F		
EBI-2024-0679				Rh	Poor appetite	Crushing and drenching: same preparation administered orally	O	F		
